# Revealing potential anti-fibrotic mechanism of Ganxianfang formula based on RNA sequence

**DOI:** 10.1186/s13020-022-00579-7

**Published:** 2022-02-18

**Authors:** Zongyi Liu, Huanyu Xiang, Dejuan Xiang, Shuang Xiao, Hongyan Xiang, Jing Xiao, Hong Ren, Peng Hu, Huabao Liu, Mingli Peng

**Affiliations:** 1 Key Laboratory of Molecular Biology for Infectious Diseases (Ministry of Education), Institute for Viral Hepatitis, Department of Infectious Diseases, The Second Affiliated Hospital, Chongqing Medical University, Chongqing, 400010 China; 2 Department of Liver Diseases, Chongqing Traditional Chinese Medicine Hospital, Chongqing, 400021 China

**Keywords:** Ganxianfang formula, Traditional Chinese medicine, RNA-seq, Liver fibrosis, Chemokine signaling pathway

## Abstract

**Background:**

Ganxianfang (GXF) formula as a traditional Chinese medicine (TCM) is used for liver fibrosis in clinical practice while its mechanism is unclear. The aim of this study is to explore the molecular mechanism of GXF against CCl_4_-induced liver fibrosis rats.

**Methods:**

Detected the main compounds of GXF by UPLC-MS/MS. Evaluated the efficacy of GXF (1.58, 3.15, 4.73 g/kg/day) and Fuzheng Huayu (FZHY, positive control, 0.47 g/kg/day) through serum alanine aminotransferase (ALT), aspartate aminotransferase (AST) levels and histopathological changes. Explored the underlying mechanisms by integrating our total liver RNA sequencing (RNA-seq) data with recent liver single-cell sequencing (scRNA-seq) studies. Verified potential pharmacodynamic substances of GXF by hepatic stellate cell (HSC)-T6 line.

**Results:**

Main compounds were identified in GXF by UPLC-MS/MS, including baicalin, wogonoside and matrine etc. With GXF-high dose treatment, the elevation of ALT and AST induced by CCl_4_ were significantly reduced, and the protective effect of GXF-high dose treatment was better than FZHY. Liver histopathological changes were alleviated by GXF-high dose treatment, the ISHAK scoring showed the incidence of liver cirrhosis (F5/F6) decreased from 76.5 to 55.6%. The results of liver hydroxyproline content were consistent with the histopathological changes. RNA-seq analysis revealed the differential genes (DEGs) were mainly enriched in ECM-receptor interaction and chemokine signaling pathway. GXF effectively inhibited collagen deposition and significantly downregulated CCL2 to inhibit the recruitment of macrophages in liver tissue. Integrating scRNA-seq data revealed that GXF effectively inhibited the expansion of scar-associated Trem2^+^CD9^+^ macrophages subpopulation and PDGFRα^+^PDGFRβ^+^ scar-producing myofibroblasts in the damaged liver, and remodeled the fibrotic niche via regulation of ligand-receptor interactions including TGFβ/EGFR, PDGFB/PDGFRα, and TNFSF12/TNFRSF12a signaling. In vitro experiments demonstrated that baicalin, matrine and hesperidin in GXF inhibited the activation of hepatic stellate cells.

**Conclusions:**

This study clarified the potential anti-fibrotic effects and molecular mechanism of GXF in CCl_4_-induced liver fibrosis rats, which deserves further promotion and application.

**Supplementary Information:**

The online version contains supplementary material available at 10.1186/s13020-022-00579-7.

## Background

Liver fibrosis is a histological change caused by acute or chronic liver diseases, such as hepatitis virus infection, autoimmune liver diseases, and alcoholic or non-alcoholic fatty liver disease [1]. The main pathological feature of liver fibrosis is the excessive deposition of extracellular matrix (ECM) component, which disrupts normal liver structure and function, resulting in the decompensation of liver cirrhosis and the occurrence of liver cancer [2, 3]. Therefore, it is important to effectively reverse liver fibrosis. At present, there is no specific medicine for the treatment of liver fibrosis except for the etiological treatment. Due to the complicated pathological mechanism of liver fibrosis, drugs developed for a single target are difficult to be effective in clinical practice [4]. Studies in recent decades have shown that traditional Chinese medicine (TCM) treatments have shown curative advantages in the field of prevention and treatment of liver fibrosis [5–7]. In clinical practice in China, several traditional Chinese medicines such as Fuzheng Huayu, Biejia Ruangan tablet and Anluo Huaxian pill are widely used in anti-fibrosis treatment, and Fuzheng Huayu has been included in the guideline for the diagnosis and treatment of liver fibrosis with integrated traditional Chinese and western medicine [8–10]. Therefore, traditional Chinese medicines have a broad prospect for the treatment of liver fibrosis.

Ganxianfang (GXF) formula is obtained by summarizing the traditional Chinese medicine prescriptions from "Jin Gui Yao Lue". Combined with the clinical experience of Professor Huabao Liu from Chongqing Traditional Chinese Medicine Hospital, GXF has been further optimized. This prescription contains a total of five herbs: Huang Qin (Scutellariae Radix), Ku Shen (Sophorae Flavescentis Radix), Fang Feng (Saposhnikoviae Radix), Chen Pi (Citrus Reticulata) and Xi Yang Shen (Panacis Quinquefolii Radix). Modern pharmacological studies indicated that Scutellariae Radix and Saposhnikoviae Radix in GXF have been reported to attenuate inflammation [11, 12]. In addition, Sophorae Flavescentis Radix has also been reported to have anti-fibrotic effects [13]. GXF has been commonly used in Chongqing region and exerts remarkable clinical effects on hepatic protection. After a large number of clinical observations, we found that 70.3% of patients with liver cirrhosis, caused by hepatitis B and alcoholic liver disease, and Child–Pugh class A or B can be reversed after GXF treatment. For patients with Child–Pugh class C, GXF treatment can stabilize liver function and reduce portal hypertension. A large cohort of clinical study is currently being established to further scientifically confirm its anti-fibrosis efficacy. Although GXF has a great anti-fibrotic potential, the complex chemical compositions have blocked the systematic understanding of the material basis of classic TCM formulas. More importantly, it is difficult to progress its use on a wider scale due to its unclear molecular mechanism.

**Fig. 1:**
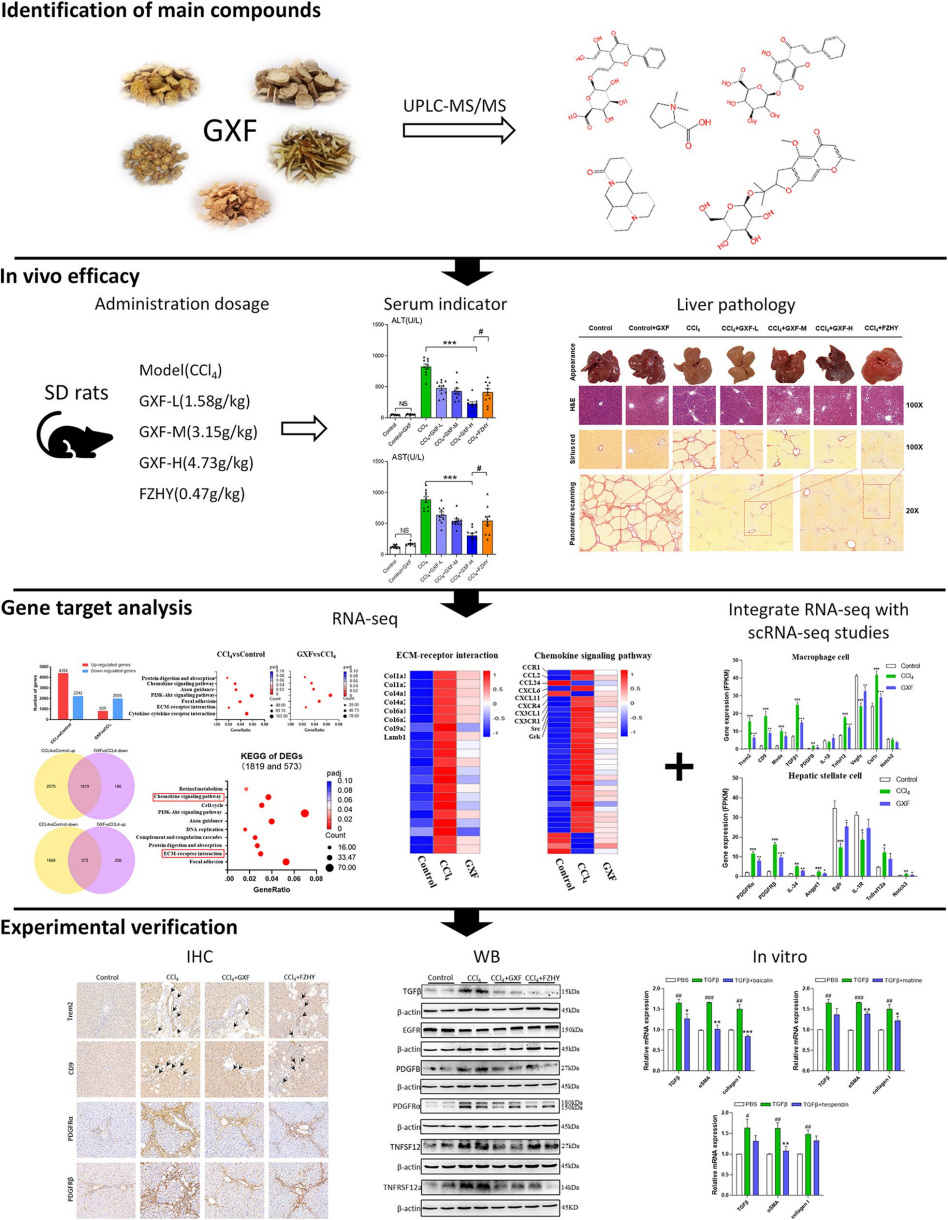
Workflow of this study

The point of this research is to verify the efficacy of GXF and explore the potential molecular mechanism on the premise of identifying the material basis of GXF through UPLC-MS/MS. The rat model involved an artificial liver injury was induced by CCl_4_, a selective hepatotoxic drug, which can trigger inflammation and fibrosis. This model simulated clinical liver fibrosis in morphology and pathophysiology [14–16]. The clinical dosage of GXF is 30 g/person/day, and used this as the standard to set the middle dose (3.15 g/kg) for rats and set high (4.73 g/kg) and low dose (1.58 g/kg) in this study. We explored the potential mechanism of GXF with our RNA sequencing (RNA-seq) data combined with recent scRNA-seq studies [17–19]. With the supplementary demonstration of basic experiments, the clinical application of GXF could be better promoted. Figure 1 shows the flow chart of the whole study.

## Materials and methods

### Reagents

Antibodies to CCL2, EGFR, PDGFRα, PDGFB and TNFRSF12a were purchased from Abcam China; CCR2, TGFβ and TNFSF12 were purchased from Affinity Biologicals, Inc; PDGFRβ and β-actin were purchased from Cell Signaling Technology; CD9 was purchased from Proteintech Group, Inc; whereas Trem2 was purchased from ABclonal Technology. Trizol reagents were purchased from Gibco BRL (NY, USA). Carbon tetrachloride, chloroform, methanol, and so forth were obtained from the Center of Equipment and Reagent (Chongqing Medical University). All the reagents were of analytical quality. FZHY was obtained from Shanghai HuangHai Pharmaceutical Co., Ltd (Shanghai, China).

### Preparation of GXF

GXF formula was obtained from Chongqing Traditional Chinese Medicine Hospital. Specifically, GXF was composed of Huang Qin (Scutellariae Radix), Ku Shen (Sophorae Flavescentis Radix), Fang Feng (Saposhnikoviae Radix), Chen Pi (Citrus Reticulata) and Xi Yang Shen (Panacis Quinquefolii Radix), which were purchased from Beijing Kangrentang Pharmaceutical Company (China). According to the traditional water extraction method, the five herbs were soaked in water at a ratio of 6:4:2:1:1 for 60 min and then boiled. The extraction was filtered and concentrated for subsequent experiments.

### UPLC-MS/MS analysis of GXF

GXF formula was analyzed using U3000 UPLC system equipped with Q Exactive Plus Orbitrap high-resolution mass spectrometer (Thermo Fisher Scientific). Chromatographic separation was performed on a ACQUITY UPLC HSS T3 column (2.1 × 100 mm, 1.8 μm, Waters). The column temperature was set at 35 ℃. The flow rate was 0.3 mL/min. The mobile phase consisted of deionized water with 0.1% formic acid (A) and acetonitrile with 0.1% formic acid (B). The mass spectrometer analysis was conducted in both positive and negative ion modes. The scan mass ratio was within the mass range of m/z 50–1500.

The analysis data were analyzed by Compound Discoverer software 3.2 (Thermo Fisher Scientific). The mass deviations of characteristic peak element matching, molecular formula prediction and isotope distribution matching were all set to within 5 ppm. Chemical identification was based on chromatographic elution behavior, mass spectrometry fragment patterns and mass spectrometry databases (MZ cloud, MZ vault).

### Animals and experiment designs

**Fig. 2:**
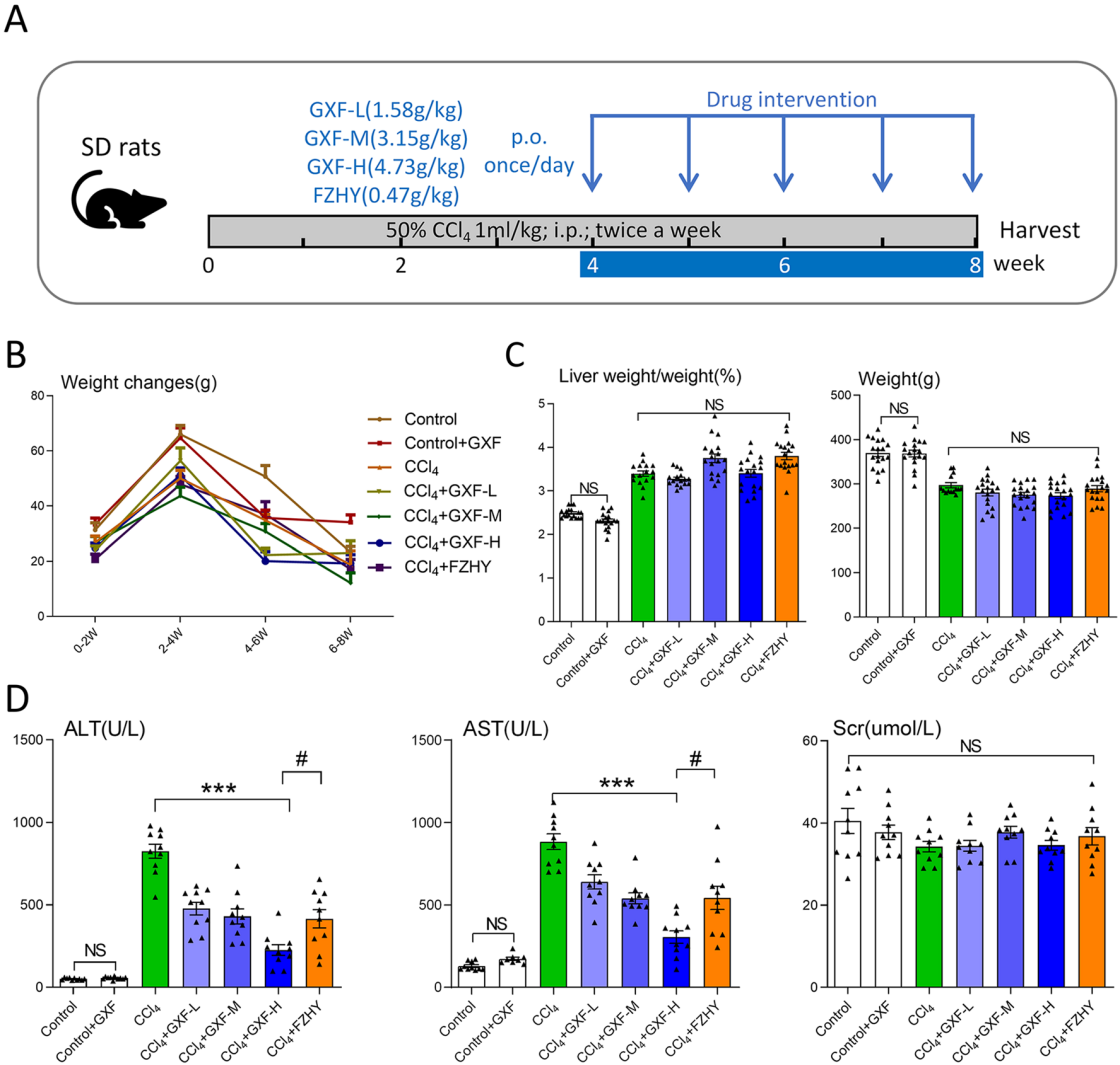
Biochemical parameters for rats evaluated in this study. **A** Schematic representation showing the experimental design and timeline of rat model. **B** Changes in body weight every 2 weeks. **C** Liver weight/weight ratio and weight in different groups by the end of week 8 (n = 20). **D** Levels of serum ALT, AST and Scr (n = 10). The data are presented as means ± SEM; NS, no significant difference. ^***^p < 0.001 compared with CCl_4_ group; ^#^*p* < 0.05 compared with FZHY group

Animals were purchased from the Laboratory Animal Center of Chongqing Medical University [SCXK(Yu)2018-0003]. The animal experiments were reviewed and approved by the Ethics Committee of Chongqing Traditional Chinese Medicine Hospital (2018-ky-48) and conformed to the Guidelines for the Care and Use of Laboratory Animals. 140 male Sprague–Dawley rats weighing 140 ~ 160 g were housed in a room at 22 ℃ with a 12-h light/dark cycle and free access to food and water. All rats acclimatized for 1 week before experiments. Liver fibrosis was induced by intraperitoneal (i.p.) injection of CCl_4_ dissolved in olive oil [1:1(v/v)] at a dose of 0.1 mL/100 g body weight (BW) twice-weekly for 8 weeks as shown in Fig. 2A. Rats were randomly divided into seven groups (n = 20 per group): (1–2) Control and GXF high-dose control groups were i.p. injected with olive oil and given 0.9% NaCl or 4.73 g/kg GXF daily respectively (p.o.); (3) CCl_4_ group; (4–6) CCl_4_ + GXF group (1.58, 3.15, 4.73 g/kg respectively, p.o.); (7) CCl_4_ + FZHY group (0.47 g/kg, p.o.). GXF and FZHY applied once daily during 5–8 weeks. Rats were anesthetized and sacrificed 48 h after the final CCl_4_ (or olive oil) injection. The livers were frozen and stored in liquid nitrogen or fixed in 4% buffered paraformaldehyde until further analysis. Serum was stored at − 80 ℃ for further analysis.

### Detection of serum ALT, AST, Scr and CCL2 levels

Serum alanine aminotransferase (ALT), aspartate aminotransferase (AST) and Scr were measured by routine enzymatic assays kits (Maccura, Chengdu, China) using HITACHI Clinical Analyzer 7600 (Hitachi, Tokyo, Japan). The detection of these indicators was completed at the Clinical Laboratory Center of the Second Affiliated Hospital of Chongqing Medical University. The serum CCL2 level was measured by sandwich enzyme-linked immunosorbent assays (ELISA) using an ELISA kit according to the manufacturer’s instructions (Raybiotech, GA, USA).

### Determination of hepatic hydroxyproline

Hydroxyproline (Hyp) contents of liver tissues were determined using an alkaline hydrolysis method described by the hydroxyproline test kit (Jiancheng Biotech, Jiangsu, China).

### Fibrosis grade assessment

Grading of liver fibrosis was assessed by collagen stained with Sirius red, all rat livers treated for 8 weeks were evaluated. The fibrosis stage was evaluated according to the Ishak grading system on a scale from F0 to F6, higher score indicates greater fibrosis. F0: No fibrosis, F1: Fibrous expansion of some portal areas, F2: Fibrous expansion of most portal areas, F3: Fibrous expansion of most portal areas with portal to portal bridging, F4: Fibrous expansion of portal areas with marked bridging, F5: Marked bridging with occasional nodules (incomplete cirrhosis), F6: Cirrhosis, probable or definite [20]. Histological assessment was performed by two clinical pathologists without knowledge of the experimental design.

### RNA-sequencing and bioinformatic analyses

RNA-Seq analysis was based on biological replicates with 8 rats in each experimental group. Library construction, RNA-seq and data analysis were performed by Novogene (Beijing, China). Total RNA was extracted from rat liver tissues with Trizol reagent (Invitrogen, USA). All samples passed RNA quality control examined by Agilent Bioanalyzer 2100, and mRNA libraries were sequenced using a 2 × 150 bp paired-end method by illumina novaseq 6000. The raw data were trimmed, filtered and qualified using FASTX (http://hannonlab.cshl.edu/fastx_toolkit/). Clean data was aligned to rat reference genome (rn5) using Hisat2 [21]. Feature Counts v1.5.0-p3 [22] was used to count the reads numbers mapped to each gene. And then FPKM [23] of each gene was calculated based on the length of the gene and reads count mapped to this gene. DESeq2 [24] was used to estimate the significance of differential genes (DEGs) between any two experimental groups according to the criteria of |log2(fold change) |> 1 and p-adjust < 0.05. KEGG is a database resource for understanding high-level functions and utilities of the biological system (http://www.genome.jp/kegg/). We used cluster Profiler R package to test the statistical enrichment of differential expression genes in KEGG pathways.

### Immunohistochemistry

Immunohistochemistry for Trem2, CD9, PDGFRα and PDGFRβ were performed using commercial kit (ZSGB-BIO, China) according to the manufacturer’s instructions. In brief, deparaffinized and blocked 5 μM sections were incubated with anti-Trem2 antibody (1:100, A10482), anti-CD9 antibody (1:3000, 60232–1-Ig), anti-PDGFR alpha (PDGFRα) antibody (1:200, ab203491) and anti-PDGFR β antibody (1:100, 3169). The sections were then further incubated with the HRP-conjugated secondary antibody for 1 h at room temperature. After washing with PBS, 3,3'-adiaminobenzidine tetrahydrochloride (DAB) coloration signal was used. Images were acquired on a Slide scanning system Pannoramic MIDI II (3D HISTECH). Certified 3D HISTECH CaseViewer and imageJ software were applied to analyze the results.

### RT-qPCR and western blot analysis

Total RNA was extracted from HSC-T6 cells or rat liver tissues with RNApure total RNA fast isolation kit (BioTeke, China) following the manufacturer’s instructions. The cDNAs were synthesized with the commercial kit (Takara, Japan). Gene expressions were measured by qPCR with CFX Connect™ Real-Time PCR System (Bio-Rad, USA). GAPDH was used as an internal control and the relative expression levels of mRNA were calculated using the 2^−ΔΔCt^ method. The primer pairs used in the experiments were listed in Additional file 1: Table S1.

Total protein was extracted from pieces of liver tissues using whole cell lysis assay (KeyGen Biotech, Jiangsu, China). Protein concentration was determined by the BCA Protein Assay Kit (KeyGen Biotech, Jiangsu, China). Quantified proteins were separated on SDS-PAGE and transferred to PVDF membranes (Millipore Corporation, USA). After blocking, membranes were incubated with primary antibodies against CCL2 (1:1000, ab7202), anti-CCR2 (1:500, DF2711), TGFβ (1:1500, AF1027), PDGF-B (1:1500, ab178409), TNFSF12 (1:500, DF7444), EGFR (1:2000, ab52894), PDGFRα (1:1000, ab203491) and TNFRSF12a (1:1500, ab109365) at 4 °C overnight. The anti-β-actin (1:2000, 4970L) was probed as an internal control. Then, membranes were washed with TBST, and incubated with secondary antibodies for 2 h at room temperature. Protein bands were visualized by using ECL (Advansta, USA). Levels of target protein band densities were analyzed with ChemicDoc™ MP Imaging System (Bio-Rad, USA).

### Cell culture, viability assay and treatment

HSC-T6 cells were cultured in DMEM (supplemented with 10% FBS and 1% P/S) at 37 ℃ in a 5% CO_2_ humidified atmosphere. Cell viability of HSC-T6 was assessed by cell counting kit-8 (CCK-8) assay (APExBIO Technology, USA). HSC-T6 cells (1 × 10^4^ cells per well) were seeded in 96-well plates, then cells were treated with different doses of baicalin, matrine or hesperidin (6.25, 12.5, 25, 50, 100 and 200 µM) for 24 h. Then, a total 10 µL of CCK-8 reagent was added to each well. After incubation for 1 h, the absorbance at 450 nm of each well was measured by a microplate reader. For TGFβ1-induced HSCs activation, HSCs were stimulated with 10 ng/mL TGFβ1 (Peprotech, USA) for 24 h. For treatment groups, HSCs were treated with TGFβ1 (10 ng/mL) supplemented with 50 μM baicalin, matrine or hesperidin (MCE, USA) for 24 h.

### Statistical analysis

GraphPad Prism 9.0 (GraphPad Software Inc., California, USA) and SPSS 25.0 (SPSS Inc., IL, USA) were used for statistical analysis. Continuous variables are presented as the mean ± SEM. The differences between two groups were assessed with Student's t-test. Statistical differences between groups were evaluated by one-way analysis of variance (ANOVA), and Ishak scores were performed with rank-sum test. A P-value of p < 0.05 was considered statistically significant.

## Results

### Identification of compounds in GXF

UPLC-MS/MS was employed to identify the main 10 compounds in GXF formula: baicalin, wogonoside, matrine, stachydrine, 5-*O*-Methylvisammioside, hesperidin, ursodeoxycholic acid, amygdalin, sophocarpine and paeoniflorin. The detailed results were presented in Table 1 (Additional file 2: Table S2).

**Table 1:** Main compounds of GXF

Compound	Chemical structure	Formula	Rt (min)	Relative content (%)
Baicalin		C_21_H_18_O_11_	26.02	17.47
Wogonoside		C_22_H_20_O_11_	28.02	9.76
Matrine		C_15_H_24_N_2_O	20.07	6.43
Stachydrine		C_7_H_13_NO_2_	1.70	3.33
5-O-Methylvisammioside		C_22_H_28_O_10_	24.25	2.99
Hesperidin		C_28_H_34_O_15_	24.67	2.75
Ursodeoxycholic acid		C_24_H_40_O_4_	38.47	2.41
Amygdalin		C_20_H_27_NO_11_	21.77	2.11
Sophocarpine		C_15_H_22_N_2_O	20.23	1.95
Paeoniflorin		C_23_H_28_O_11_	22.89	1.56

### GXF improved CCl_4_-induced liver function in rats

The process of animal experiment is shown in Fig. 2A. The body weight of rats was weighted once a week during the experiment process. The weight changes of the rats every 2 weeks were calculated, although the weight changes of the control group and the GXF control group were greater than the others, there was no statistical difference in the weight changes between the CCl_4_ group and the treatment groups (Fig. 2B). The body weight and liver weight of rats at the eighth week showed that GXF had no effect on the weight and liver weight/body weight ratio (Fig. 2C). Serum ALT, AST and Scr levels were used to assess liver and renal function. There was no significant difference in serum Scr level within each group, indicating that GXF had no effect on the renal function. Compared with control group, the serum ALT and AST levels of control drug group had no significant change, while CCl_4_ group had a significantly increase (*p* < 0.001). Further, the liver injury induced by CCl_4_ was reversed to varying degrees by GXF (1.58, 3.15, 4.73 g/kg/day) and FZHY (0.47 g/kg/day). Among the treatment groups, the serum ALT and AST levels of the GXF high-dose (4.73 g/kg/day) group had the most significant decrease compared with the CCl_4_ group (*p* < 0.001). In addition, the ALT and AST levels of the GXF high-dose group were lower than FZHY group (*p* < 0.05), indicating that its protective effect on liver is better than FZHY (Fig. 2D).

### GXF alleviated liver histopathological changes

**Fig. 3:**
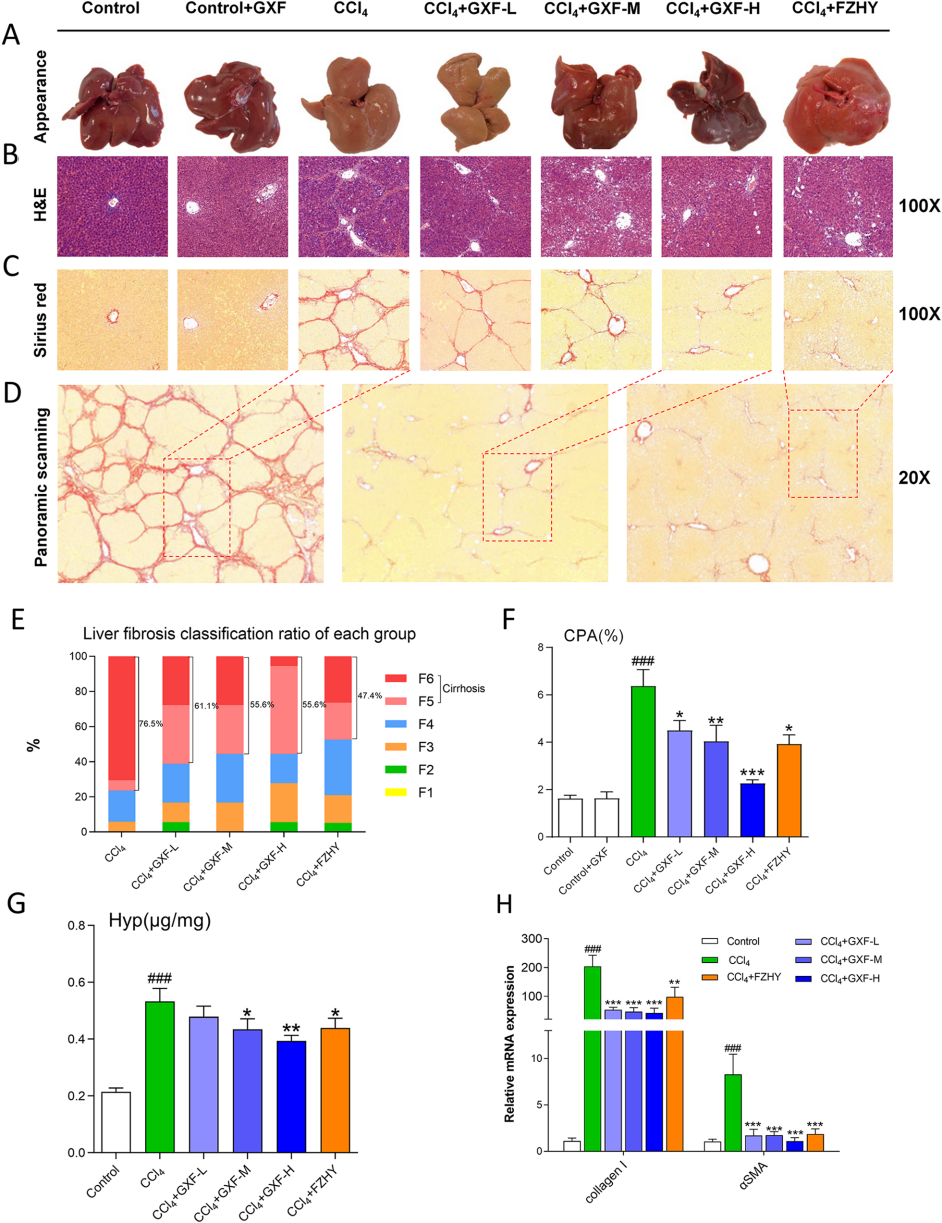
GXF alleviated CCl_4_-induced liver injury and hepatic fibrosis in rats. **A** Representative photos of liver appearance from different groups. **B** H&E, **C** Sirius red and **D** panoramic scanning of rat liver tissues. **E** Ishak stage classification of liver fibrosis (n = 20). **F** The CPA of Sirius red (n = 20). **G** Content of liver Hyp (n = 20). **H** mRNA expression of collagen I and α-SMA (n = 4). The data are presented as means ± SEM. ^###^*p* < 0.001 compared with control group; *p < 0.05, **p < 0.01, ***p < 0.001 compared with CCl_4_ group

We observed that the liver surface of CCl_4_ and GXF low-dose groups were significantly rougher compared with the control group, whereas that of GXF high-dose and FZHY groups were significantly improved (Fig. 3A). HE staining showed that CCl_4_ caused collagen deposition, structural destruction and inflammatory cell infiltration in liver lobules and portal veins, while GXF high-dose and FZHY treatment alleviated these histopathological damages (Fig. 3B). Similarly, Sirius red staining showed that the collagen deposition in the CCl_4_ group was significantly increased compared with the control group, and collagen deposition could be reduced after treatment (Fig. 3C). The reduction of collagen in GXF high-dose and FZHY groups were the most significant, which could be more intuitively reflected by the panoramic scanning (Fig. 3D). According to the analysis of the ISHAK scoring standard, the incidence of liver cirrhosis in both GXF medium-dose and high-dose decreased from 76.5 to 55.6%. Although the F5/F6 scores of FZHY group accounted for the lowest proportion, the F6 scores of GXF high-dose group accounted for the lowest (Fig. 3E). The CPA and Hyp content of GXF high-dose group were the lowest and significantly lower than CCl_4_ group (*p* < 0.001 or *p* < 0.01) (Fig. 3F, G). Finally, the relative mRNA expression of collagen I and α-SMA in liver tissues of each treatment group were significantly lower than that of CCl_4_ group (*p* < 0.001 or *p* < 0.01) (Fig. 3H).

### KEGG analysis revealed DEGs enriched in chemokine signaling pathway

**Fig. 4:**
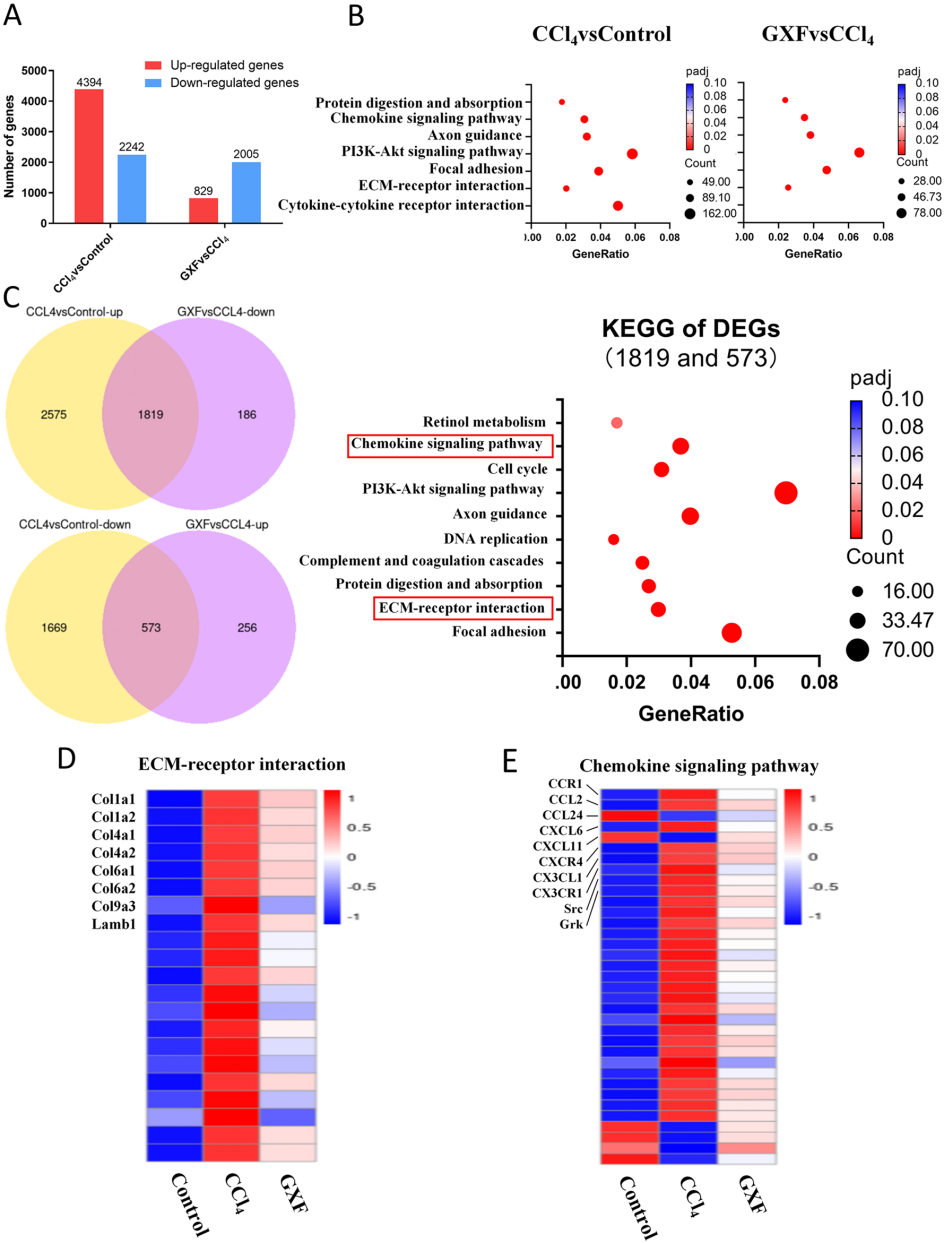
RNA sequencing analysis. Liver tissues were from control rats(n = 8), model rats (CCl_4_-treated, n = 8) and GXF rats (CCl_4_ and 4.73 g/kg dose treated, n = 8). **A** Number of DEGs by RNA-seq of total liver mRNA. Genes upregulated or downregulated by more than twofold and *p* < 0.05 were shown in red and blue, respectively. **B** Significant KEGG pathways of compared groups as shown. **C** KEGG pathways of overlapped DEGs induced by GXF and CCl_4_, and important pathways were in red box. **D** Heatmap represented the gene expression of ECM-receptor interaction. Genes related were indicated. **E** Heatmap represented the gene expression of Chemokine signaling pathway. Genes related were indicated

To investigate the molecule mechanisms underlying the protective effect of GXF against CCl_4_-induced liver fibrosis in rats, hepatic gene expression profiles of control, CCl_4_ and GXF high-dose group were detected by RNA-seq (n = 8). 6636 DEGs were identified in CCl_4_ group compared with control group, 4394 genes of which were up-regulated and 2242 were down-regulated. Similarly, 2384 DEGs were identified in GXF high-dose group compared to CCl_4_ group, 829 genes of which were up-regulated and 2005 were down-regulated (|Foldchange|> 2, p-adjust < 0.05) (Fig. 4A). The KEGG pathway analyses of the difference genes between CCl_4_ and control group and that between GXF and CCl_4_ group were showed in Fig. 4B. 2392 genes were obtained by intersecting the DEGs between the CCl_4_ and control group and the DEGs between GXF and CCl_4_ group, and the KEGG analyses of these genes were showed in Fig. 4C (Additional file 3: Table S3). The results showed that these differential genes could be significantly enriched in the ECM-receptor interaction pathway, suggesting that GXF treatment could effectively inhibit collagen deposition, and the heat map results of this pathway revealing that GXF could significantly down-regulate the expression of Col1a1, Col1a2, Col4a1 and Col6a1 (Fig. 4D). In addition, the DEGs could also be significantly enriched in the chemokine signaling pathway. The heat map showed that GXF could significantly inhibit the expression of some chemokine-related genes such as CCL2, CXCL6 and CX3CL1 (Fig. 4E), suggesting that GXF may alleviate CCl_4_-induced liver fibrosis by inhibiting the recruitment of related immune cells.

### GXF down-regulated CCL2 both in liver tissues and peripheral blood

**Fig. 5:**
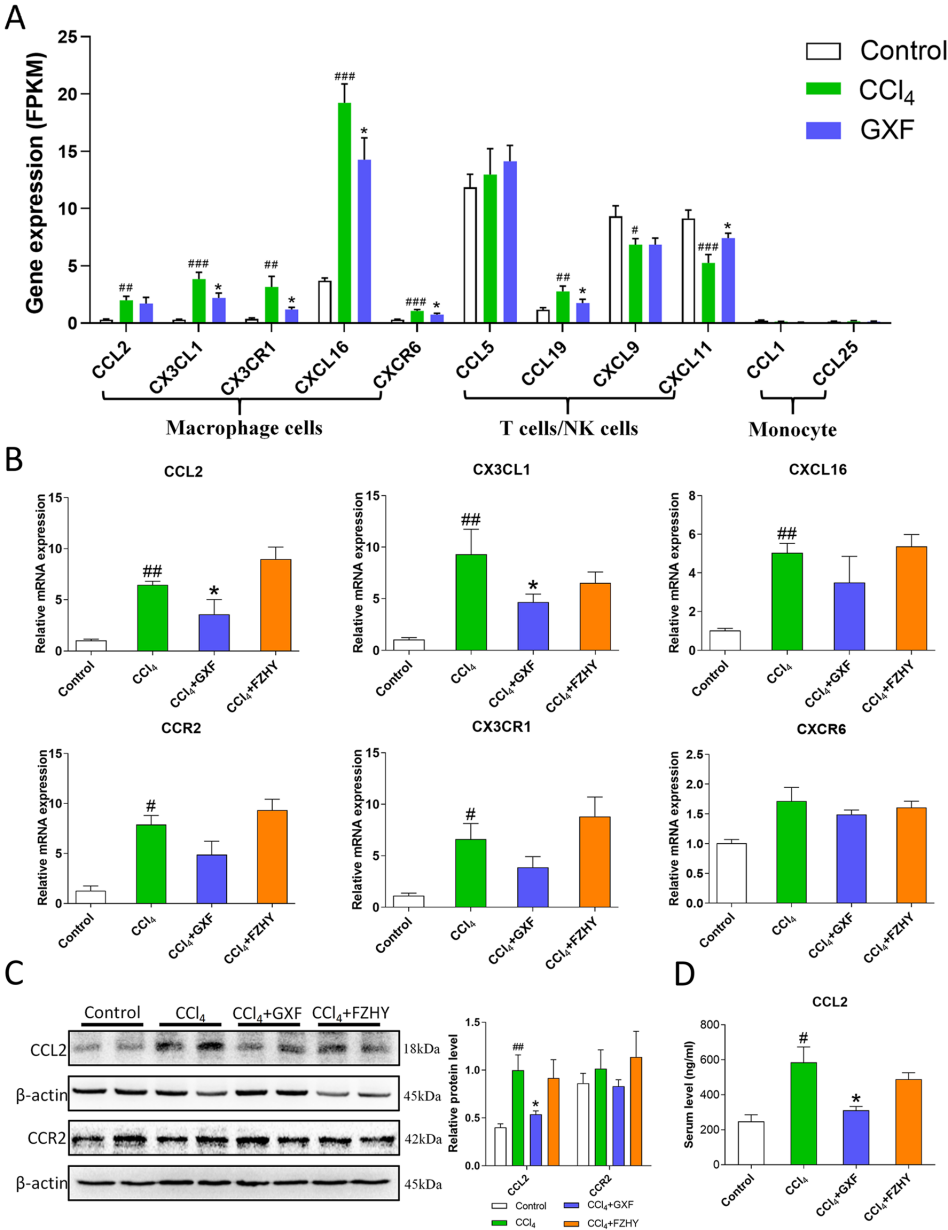
GXF down-regulated genes related to macrophages. **A** The expression of genes enriched in chemokine pathway. Gene expression level was measured in FPKM value. **B** The mRNA expression of CCL2, CCR2, CX3CL1, CX3CR1, CXCL16 and CXCR6 were evaluated by RT-qPCR. **C** The protein levels of CCL2 and CCR2 were evaluated by western blot. **D** Serum CCL2 level was evaluated by Elisa. The data are presented as means ± SEM (n = 4). ^#^*p* < 0.05, ^##^*p* < 0.01 compared with control group; *p < 0.05 compared with CCl_4_ group

According to the RNA-seq results, the expression levels of genes enriched in chemokine pathway were summarized, which showed in Fig. 5A. Between the CCl_4_ group and GXF group, there was no significant difference in expression levels of genes related to the recruitment of T cells and monocyte. However, compared with the CCl_4_ group, GXF treatment could significantly inhibit the expression of genes which related to macrophages such as CCL2, CX3CL1, CX3CR1, CXCL16 and CXCR6 (*p* < 0.05). The relative mRNA expression of CCL2, CCR2, CX3CL1, CX3CR1, CXCL16 and CXCR6 in liver tissues was detected by RT-qPCR. As shown in Fig. 5B, CCl_4_ intervention could significantly increase the expression of these genes, but it could be suppressed by GXF, of which CCL2 was the most significant (*p* < 0.05). Compared with GXF, FZHY had no obvious inhibitory effect on these genes. The western blot results showed that the protein expression of CCR2 did not differ between the groups, but the expression of CCL2 in the CCl_4_ group was significantly higher than that in the control group, which could be reduced after GXF treatment (*p* < 0.05) (Fig. 5C). In addition, the expression of CCL2 in serum was consistent with that in liver tissues (Fig. 5D). Therefore, GXF may delay the progression of liver fibrosis by inhibiting the recruitment of macrophages from peripheral blood to liver tissues.

### GXF remodeled the fibrotic niche

**Fig. 6:**
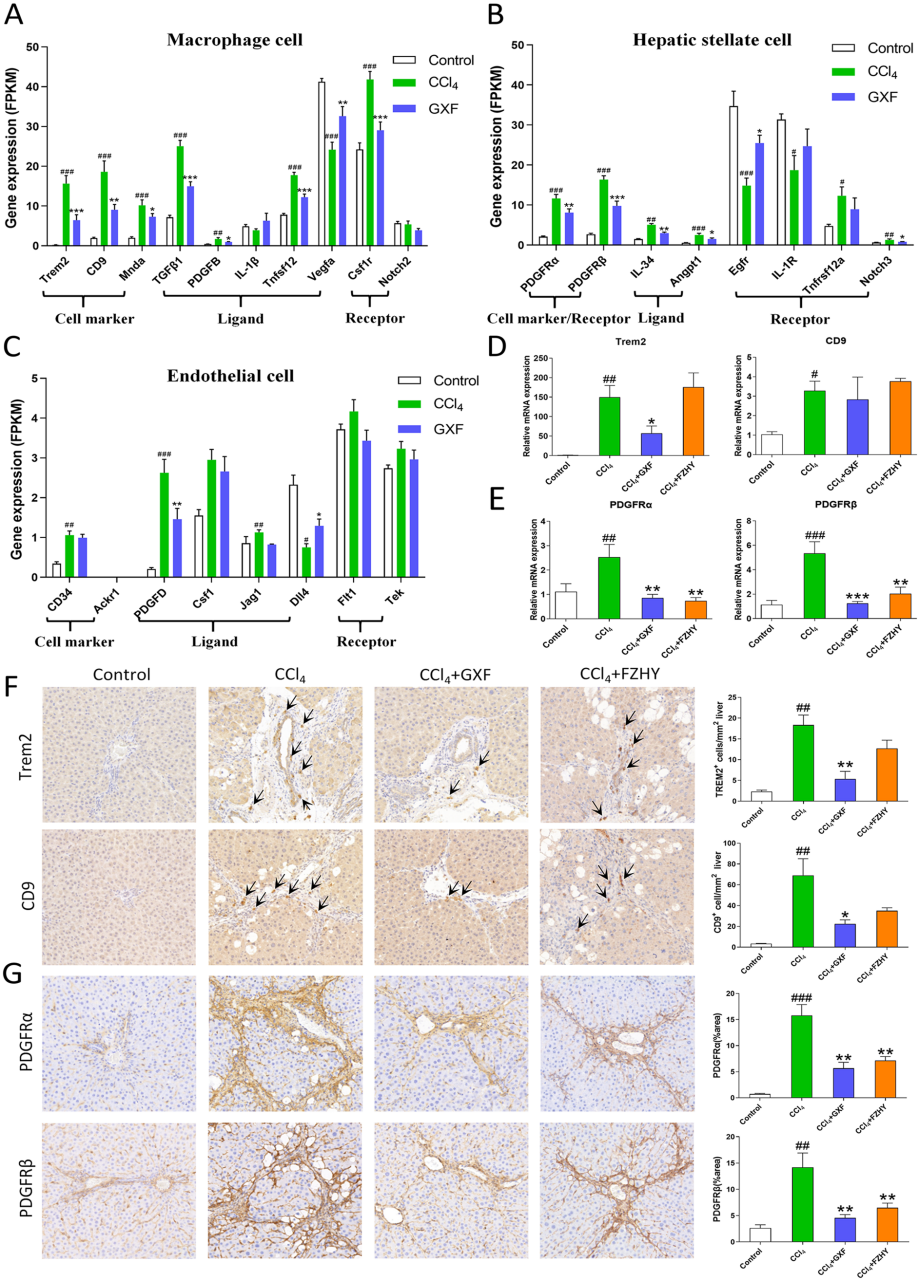
GXF reduced the proliferation and activation of SAMacs and SAMes. **A-C** scRNA-seq combined with RNA-seq to analyze the expression of genes in macrophages, endothelial cells and HSCs. Gene expression level was measured in FPKM value. **D** The mRNA expression of Trem2 and CD9 were evaluated by RT-qPCR. **E** The mRNA expression of PDGFRα and PDGFRβ were evaluated by RT-qPCR. **F** Immunohistochemical staining of liver Trem2 and CD9 levels (× 200). **G** Immunohistochemical staining of liver PDGFRα and PDGFRβ levels (× 200). The data are presented as means ± SEM (n = 4). ^#^*p* < 0.05, ^##^*p* < 0.01, ^###^*p* < 0.001 compared with control group; *p < 0.05, **p < 0.01, ***p < 0.001 compared with CCl_4_ group

Single-cell sequencing (scRNA-seq) studies have already provided a wealth of novel insights into cellular heterogeneity and the interactome present across the different cell types in liver fibrosis progression [18]. The interaction between macrophages, stellate cells and endothelial cells is closely related to liver fibrosis [19]. To interrogate how GXF attenuates fibrosis and to deepen our understanding of the mechanism, we integrated our total liver RNA-seq data with recent liver scRNA-seq studies. According to the RNA-seq results, the gene expression levels of genes related to scar-associated macrophages (SAMacs), scar-associated mesenchymal cells (SAMes) and scar-related endothelial (SAEndo) were summarized in Fig. 6A–C. Compared with the CCl_4_ group, RT-qPCR results showed that the unique markers of SAMacs such as Trem2 and CD9 were down-regulated by GXF (*p* < 0.05) (Fig. 6D). More intuitively, the number of Trem2^+^CD9^+^ macrophages were increased in the CCl_4_ and FZHY groups compared with the control group, while it was not observed in the GXF group (*p* < 0.05 or *p* < 0.01) (Fig. 6F). The distinct genes including PDGFRα and PDGFRβ characterized to SAMes were also obviously reduced by GXF (*p* < 0.01 or *p* < 0.001) (Fig. 6E), and this trend was highly consistent with the results of immunohistochemistry (*p* < 0.01) (Fig. 6G). Whereas, there was no obvious effect on the specific molecules of SAEndo subpopulations including Cd34, Ackr1, Csf1, Flt1 and Tek (Fig. 6C). Therefore, we speculated that GXF treatment could reduce the proliferation and activation of SAMacs and SAMes, but had little effect on SAEndo.

### GXF interfered the interaction between macrophages and stellate cells

**Fig. 7:**
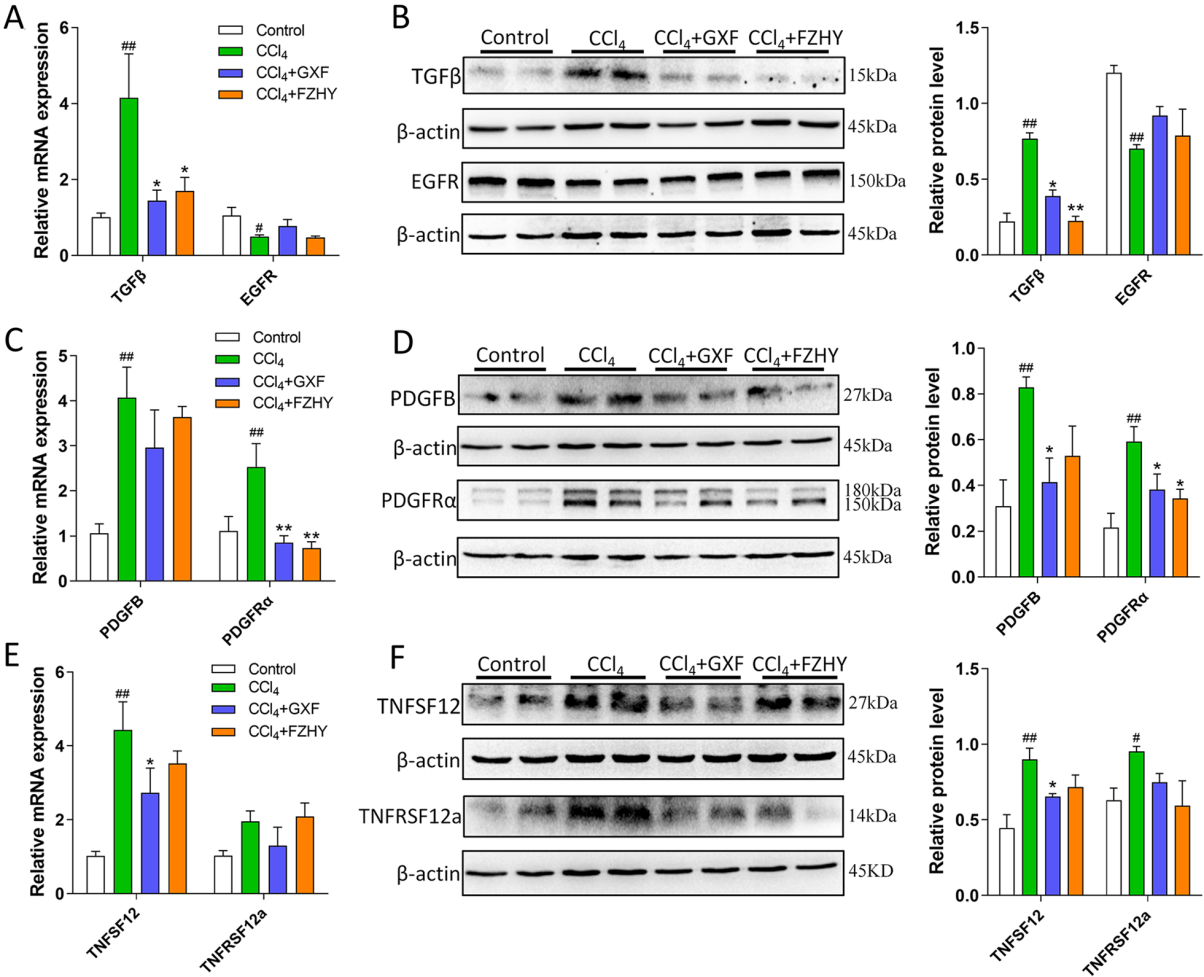
GXF interfered the interaction between macrophages and stellate cells. **A** The mRNA expression of TGFβ and EGFR were evaluated by RT-qPCR. **B** The protein levels of TGFβ and EGFR. **C** The mRNA expression of PDGFB and PDGFRα were evaluated by RT-qPCR. **D** The protein levels of PDGFB and PDGFRα. **E** The mRNA expression of TNFSF12 and TNFRSF12a were evaluated by RT-qPCR. **F** The protein levels of TNFSF12 and TNFRSF12a. The data are presented as means ± SEM (n = 4). ^#^*p* < 0.05, ^##^*p* < 0.01 compared with control group; *p < 0.05, **p < 0.01 compared with CCl_4_ group

Ligands and receptors bridge the interaction between cells. TGFβ/EGFR, PDGFB/PDGFRα and TNFSF12/TNFRSF12a are important bonds between macrophages and stellate cells. We found that GXF could effectively inhibit the expression of PDGFB and TNFSF12 on SAMacs and its cognate receptor PDGFRα and TNFRSF12a expressed on SAMes (*p* < 0.05 or *p* < 0.01) (Fig. 7C–F). Although GXF had no effect on the expression of EGFR compared with the CCl_4_ group, it could significantly inhibit the expression of TGFβ in liver tissues (*p* < 0.05) (Fig. 7A, B). In addition, western blot results showed that FZHY also had a certain inhibitory effect on the expression of the above ligands and receptors, indicating that FZHY also inhibited the activation of stellate cells by interfering the interaction between macrophages and stellate cells. Overall, these data suggested that the anti-fibrosis effect of GXF is closely related to its inhibition expansion of scar-associated Trem2^+^CD9^+^ macrophage subpopulation and PDGFRα^+^ PDGFRβ^+^ scar-producing myofibroblasts in the damaged liver, and its remodeling of the fibrotic niche through regulation ligand-receptor interactions between SAMacs and SAMes including TGFβ/EGFR, PDGFB/PDGFRα and TNFSF12/TNFRSF12a signaling.

### Verified potential pharmacodynamic substances of GXF

**Fig. 8:**
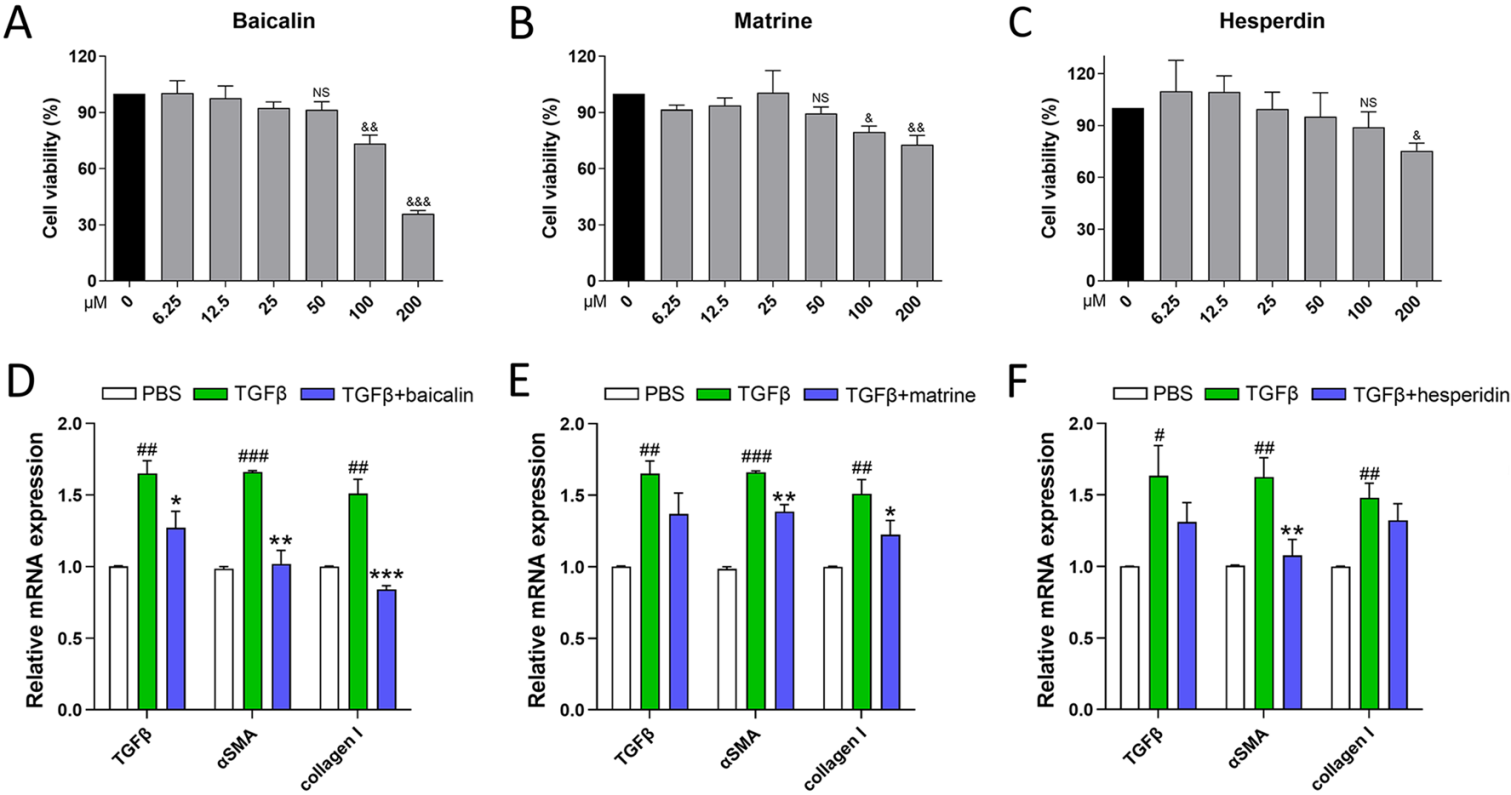
Main compounds of GXF inhibited activation of TGFβ1-induced HSCs. **A**–**C** Effects of different concentrations of baicalin, matrine and hesperidin on HSC-T6 cells viability by CCK-8 assay. **D**–**F** The mRNA expression of TGFβ, α-SMA and collagen I in TGFβ1-activated HSC-T6 cells treated with baicalin, matrine or hesperidin (50 μM). The data are presented as means ± SEM (n = 3). ^&^*p* < 0.05, ^&&^*p* < 0.01, ^&&&^*p* < 0.001 compared with control group; ^#^*p* < 0.05, ^##^*p* < 0.01, ^###^*p* < 0.001 compared with PBS group; *p < 0.05, **p < 0.01, ***p < 0.001 compared with TGFβ group

Based on the main compounds of GXF identified by UPLC-MS/MS, we assessed the potential pharmacodynamic substances on TGFβ1-activated HSC-T6 cells. Results of CCK-8 assay indicated that baicalin, matrine and hesperidin, with a concentration no higher than 50 μM, had no significant inhibitory effect on the viability of HSC-T6 cells (Fig. 8A–C). Next, activation of HSCs could be effectively reversed under the intervention of these three drugs at a concentration of 50 μM, as indicated by inhibited mRNA levels of TGFβ, α-SMA and collagen I (Fig. 8D–F). In vitro experiments demonstrated that baicalin, matrine and hesperidin inhibited the activation of HSCs, which deserves further attention.

## Discussion

**Fig. 9:**
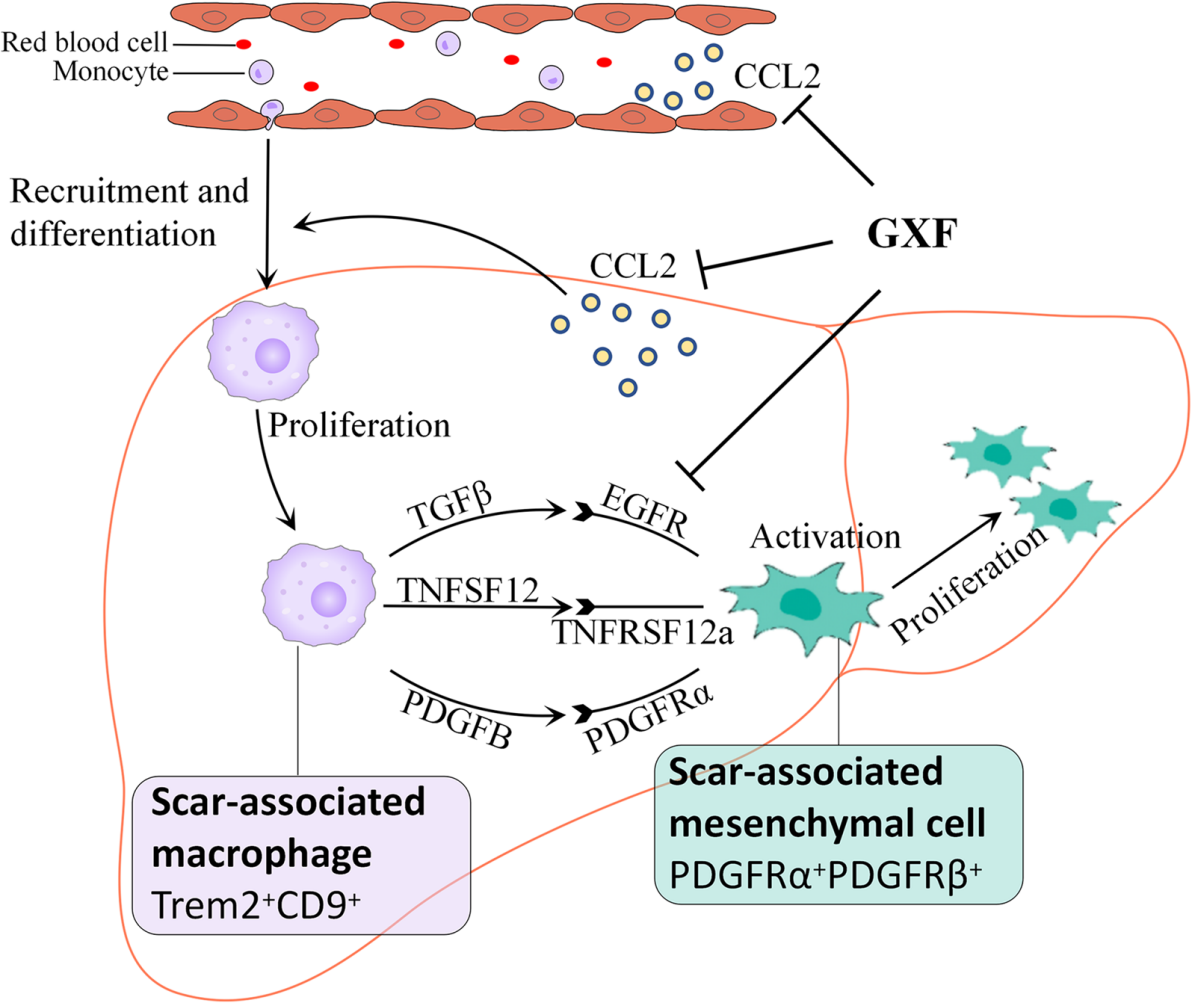
Schematic illustration of molecular mechanism of GXF in CCl_4_-induced hepatic fibrosis

As there are no effective drugs so far, traditional Chinese medicine has advantages in the treatment of liver fibrosis [25, 26]. In this study, we used UPLC-MS/MS to identify the compound composition of GXF to deepen our understanding of the material basis of classic TCM formulas. Next, we have confirmed through animal experiments that the GXF high-dose has a good therapeutic effect on CCl_4_-induced liver fibrosis, which is no less effective than FZHY. Integration of our RNA-seq analysis and recent scRNA-seq data showed that GXF significantly downregulated CCL2 to inhibit the expansion of scar-associated Trem2^+^CD9^+^ macrophages subpopulation and PDGFRα^+^PDGFRβ^+^ scar-producing myofibroblasts in the damaged liver. Mechanisms of GXF inhibiting liver fibrosis are summarized in Fig. 9.

UPLC-MS/MS provides technical support for analyzing the compound composition of traditional Chinese medicine. In this study, we identified 10 main compounds of GXF, of which baicalin, matrine, hesperidin and paeoniflorin have been reported to have anti-inflammatory or antioxidant activity. Baicalin is a flavonoid compound with antibacterial and anti-inflammatory effects [27]. It has been reported that baicalin can effectively reduce the level of alanine aminotransferase during active hepatitis [28]. Matrine is also a compound with anti-inflammatory activity, and it has been reported to have therapeutic potential in chronic bronchitis and enteritis [29, 30]. In addition, studies have shown that hesperidin and paeoniflorin have certain antioxidant activities [31, 32]. In vitro experiments proved that baicalin, matrine and hesperidin inhibited the activation of HSCs in this study. In brief, the therapeutic effect of GXF on liver fibrosis is determined by multiple active compounds.

Liver pathology analysis is the gold standard for evaluating the efficacy of anti-fibrotic drugs. The qualitative analysis of histopathological results was performed by the ISHAK scoring standard. Although the proportion of F5/F6 in GXF high-dose (55.6%) was not lower than FZHY (47.4%), but the proportion of F6 in GXF high-dose (5.6%) was the lowest among the treatment groups. In addition, the F2/F3 scores of GXF high-dose accounted for the highest proportion (27.8%), revealing that GXF high-dose had the best reversal effect on CCl_4_-induced liver fibrosis. Hydroxyproline is also an indicator of collagen deposition [33]. The hydroxyproline content of GXF high-dose (0.39 μg/mg) was lower than the CCl_4_ group (0.53 μg/mg) and the FZHY group (0.43 μg/mg), which further proved the effectiveness of GXF high-dose from a quantitative perspective. We believe that GXF high-dose may have a better delaying effect on the progression of advanced cirrhosis.

As we know, hepatic inflammation is a major contributor to the pathogenesis of almost all liver diseases [34]. Low-molecular-weight proteins called chemokines such as CCL2, CX3CL1, CXCL16 and CCL5 are the main drivers of liver infiltration by immune cells such as macrophages, neutrophils and others during an inflammatory response [35, 36]. Chemokine CCL2/CCR2 signaling plays an important role in the process of fibrosis [37–39]. CCL2 is secreted by hepatic stellate cells, hepatocytes, biliary epithelial cells and Kupffer cells. CCR2, the only known receptor for CCL2, is expressed on monocytes and macrophages in the liver [37]. Following the liver injury induced by CCl_4_, there is recruitment of circulating LY6C^hi^CCR2^+^ monocytes that differentiate into liver monocyte-derived macrophages, resulting in a huge expansion of intrahepatic macrophages [40, 41]. CXCL16 is produced by macrophages, which is a survival and maturation factor for hepatic NKT cells, resulting in an accumulation of NKT cells at sites of injury [42]. CX3CL1 is widely expressed on immune and non-immune cells, and CX3CL1/CX3CR1 signaling has been shown to promote macrophage survival [43, 44]. In this study, compared with the CCl_4_ group and the FZHY group, GXF could inhibit the expression of CCL2 in peripheral blood and liver tissue, thereby inhibiting the homing of macrophages. Although FZHY had no effect on the expression of CCL2, it could inhibit the expression of CX3CL1 and CXCR6, indicating that FZHY acted on macrophages in a different mechanism.

Single-cell transcriptome technologies are transforming our understanding of cell diversity and disease pathogenesis. Liver fibrosis involves a complex interplay between multiple non-parenchymal cell (NPC) lineages including SAMacs, SAMes and SAEndo spatially located within areas of scarring, termed the fibrotic niche [18]. The monocyte-derived macrophages (MDMs), which recruited by CCL2/CCR2 signaling, have been showed to regulate a number of aspects of liver injury, including inflammation and fibrosis [45, 46]. A distinct subpopulation of Trem2^+^CD9^+^ scar-associated macrophages (SAMacs) were identified in these MDMs by scRNAseq. The SAMacs, which expand in fibrotic livers, are spatially localized to areas of scarring (termed the fibrotic niche) and promote the activation of quiescent HSCs into PDGFRα^+^ SAMes, which proliferate and produce fibrillar collagens within the fibrotic niche of diseased livers [19]. In addition, due to the complex disease process of liver fibrosis, the interaction between different cells may be an important driving force for disease progression. Specifically, SAMacs express ligands including TGFβ, TNFSF12 and PDGFB that could signal through their cognate receptors EGFR, TNFRSF12a and PDGFRα expressed on SAMes to potentially promote mesenchymal cell activation [47–49] and proliferation [50, 51]. In this study, both GXF and FZHY could inhibit the above-mentioned ligand-receptor interactions between SAMacs and SAMes. In general, the application of multiomic approaches helps to deepen our new understanding of the therapeutic targets of TCM formulas.

## Conclusion

In this study, we identified the main compounds of GXF formula by UPLC-MS/MS. We verified that GXF had an anti-fibrosis effect by inhibiting the recruitment of macrophages and improving the microenvironment of hepatic stellate cells. In conclusion, this study reveals potential anti-fibrotic mechanism of GXF formula, and provides scientific basis for further studies and clinical applications.

## Supplementary Information

**Additional file 1: Table S1.** Primer sequences of RT-PCR.

**Additional file 2: Table S2.** Detailed information and detection map of the main compounds.

**Additional file 3: Table S3.** KEGG pathways of DEGs with opposite trends in GXF and CCl_4_ group.

## Data Availability

The datasets used and/or analyzed during the current study are available from the corresponding author on reasonable request.

## Abbreviations

GXF: Ganxianfang
TCM: Traditional Chinese medicine
CCl_4_: Carbon tetrachloride
RNA-Seq: RNA sequencing
scRNA-seq: Single-cell sequencing
ALT: Alanine aminotransferase
AST: Aspartate aminotransferase
Scr: Serum creatinine
FZHY: Fuzheng Huayu
Hyp: Hydroxyproline
CPA: Collagen proportion area
α-SMA: α-Smooth muscle actin
KEGG: Kyoto Encyclopedia of Genes and Genomes
DEGs: Differentially expressed genes
ECM: Extracellular matrix
CCL2: C–C motif chemokine ligand 2
CCR2: C–C motif chemokine receptor 2
SAMacs: Scar-associated macrophages
SAMes: Scar-associated mesenchymal cells
SAEndo: Scar-related endothelial
HSCs: Hepatic stellate cells
Trem2: Triggering receptor expressed on myeloid cells 2
PDGFRα: Platelet derived growth factor receptor alpha
PDGFRβ: Platelet derived growth factor receptor beta
PDGFB: Platelet derived growth factor subunit B
TGFβ: Transforming growth factor beta
EGFR: Epidermal growth factor receptor
TNFSF12: TNF superfamily member 12
TNFRSF12a: TNF receptor superfamily member 12a
