# Supplementary material for: Revealing potential anti-fibrotic mechanism of Ganxianfang formula based on RNA sequence
Source: Chin Med. 2022 Feb 18;17:23. doi: 10.1186/s13020-022-00579-7 (PMC8855591; doi:10.1186/s13020-022-00579-7)
Supplement: Supplementary file 1 — Additional file 1: Table S1. Primer sequences of RT-PCR. [file 13020_2022_579_MOESM1_ESM.docx]

Supplementary Table S1. Primer sequences of RT-PCR.

| **Genes** | **Forward primer** | **Reverse primer** |
| --- | --- | --- |
| Rat-Ccl2 | GCCTGTTGTTCACAGTTGCT | TGTAGTTCTCCAGCCGACTC |
| Rat-Ccr2 | TTGTTGGTGAGAAGTTCCGA | TCCCCAGTAGAAGGGGTAAA |
| Rat-Cx3cl1 | CCTGGCCGCGTTCTTTCATC | CGGGATTGGCGAGGTCATCT |
| Rat-Cx3cr1 | GAGCTTTTGCTACTTCCGCA | CGATGTTGTAAGGCGTCCAG |
| Rat-Cxcl16 | AGTGTCGCTGGAAGTTGCTA | ACTCTTGGACTGCAACTGGA |
| Rat-Cxcr6 | GATAGCATACCTTCGGGCCT | TGAGACTCCCAGACGAGAGA |
| Rat-Col1a1 | GTCAGACCTGTGTGTTCCCC | GATCGGAACCTTCGCTTCCA |
| Rat-a-SMA | GAAGAGCCACGGACAAGA | GTCCACAATGCCTGGGT |
| Rat-Trem2 | CTCCACCACCAAGACTTCTGTT | ACGTTTACCAGCAACCATCC |
| Rat-Cd9 | CTTGTCCCACGCAAGTCCAG | GTCGAACCGCAGCCATAGTC |
| Rat-Pdgfra | ATTCCCCTGCCAGACATTGA | AGTGGAACTACTGGAACCCG |
| Rat-Pdgfrb | GTCAATGTCCCTGTCCGTGT | GTGTGGGTGACAGTTTTCGC |
| Rat-Tgfb1 | CTGCTGACCCCCACTGATAC | TCGACGTTTGGGACTGATCC |
| Rat-Pdgfb | CTACCTGCGTCTGGTCAGC | AGAATGTGCTCGGGTCATGT |
| Rat-Tnfsf12 | CGAGCTATTGCAGCCCATTA | TACAGGTAGTAGAGCCCAGC |
| Rat-Tnfrsf12a | TTGCCTCTGAACCGGCAATC | GGCGTTGCCTGGTGCTT |
| Rat-Egfr | AACAACCAGAAGGGCCAAAGA | GTACGTTTTCGGACAAGCTGA |
| Rat-Gapdh | TTGCCCTCAACGACCACTTT | TGGTCCAGGGGTCTTACTCC |

The primers were designed with Primer6 verified by oligo 7.
